# Bisulfite profiling of the *MGMT* promoter and comparison with routine testing in glioblastoma diagnostics

**DOI:** 10.1186/s13148-022-01244-4

**Published:** 2022-02-18

**Authors:** Sascha Tierling, Wiebke M. Jürgens-Wemheuer, Alea Leismann, Julia Becker-Kettern, Michael Scherer, Arne Wrede, David Breuskin, Steffi Urbschat, Christoph Sippl, Joachim Oertel, Walter J. Schulz-Schaeffer, Jörn Walter

**Affiliations:** 1 Fak.NT Life Sciences, Department of Genetics/Epigenetics, Saarland University, Campus, Building A2 4, 66041 Saarbrücken, Germany; 2 Institute of Neuropathology, Medical Faculty of the Saarland University, Homburg, Germany; 3 Department of Bioinformatics and Genomics, Centre for Genomic Regulation, Barcelona, Spain; 4 Institute for Neurosurgery, Medical Faculty of the Saarland University, Homburg, Germany

**Keywords:** DNA methylation, Glioblastoma, *MGMT*, MSP, Local deep bisulfite sequencing

## Abstract

**Background:**

Promoter methylation of the DNA repair gene *O*^6^-methylguanine-DNA methyltransferase (*MGMT*) is an acknowledged predictive epigenetic marker in glioblastoma multiforme and anaplastic astrocytoma. Patients with methylated CpGs in the *MGMT* promoter benefit from treatment with alkylating agents, such as temozolomide, and show an improved overall survival and progression-free interval. A precise determination of *MGMT* promoter methylation is of importance for diagnostic decisions. We experienced that different methods show partially divergent results in a daily routine. For an integrated neuropathological diagnosis of malignant gliomas, we therefore currently apply a combination of methylation-specific PCR assays and pyrosequencing.

**Results:**

To better rationalize the variation across assays, we compared these standard techniques and assays to deep bisulfite sequencing results in a cohort of 80 malignant astrocytomas. Our deep analysis covers 49 CpG sites of the expanded *MGMT* promoter, including exon 1, parts of intron 1 and a region upstream of the transcription start site (TSS). We observed that deep sequencing data are in general in agreement with CpG-specific pyrosequencing, while the most widely used MSP assays published by Esteller et al. (N Engl J Med 343(19):1350–1354, 2000. 10.1056/NEJM200011093431901) and Felsberg et al. (Clin Cancer Res 15(21):6683–6693, 2009. 10.1158/1078-0432.CCR-08-2801) resulted in partially discordant results in 22 tumors (27.5%). Local deep bisulfite sequencing (LDBS) revealed that CpGs located in exon 1 are suited best to discriminate methylated from unmethylated samples. Based on LDBS data, we propose an optimized MSP primer pair with 83% and 85% concordance to pyrosequencing and LDBS data. A hitherto neglected region upstream of the TSS, with an overall higher methylation compared to exon 1 and intron 1 of *MGMT*, is also able to discriminate the methylation status.

**Conclusion:**

Our integrated analysis allows to evaluate and redefine co-methylation domains within the *MGMT* promoter and to rationalize the practical impact on assays used in daily routine diagnostics.

**Supplementary Information:**

The online version contains supplementary material available at 10.1186/s13148-022-01244-4.

## Background

Glioblastoma multiforme (GBM) are the most frequent human brain tumors in adults and represent the most aggressive form of tumors deriving from astroglia. With their accelerated, infiltrative, often multiple growth, and characteristic hallmarks such as necrosis, endothelial proliferation and high mitotic activity, they are graded as WHO grade IV tumors with an average survival of 15 months upon optimized standard treatment [1–4]. Anaplastic astrocytomas (grade III) are histologically slightly less aggressive lacking necrotic areas and endothelial proliferations, but patients are subjected to the same kind of treatment. Besides surgery and radiotherapy, the therapeutic decision mainly depends on the promoter DNA methylation of the DNA repair gene *O*^6^‐methylguanine‐DNA methyltransferase (*MGMT*). Methylation-induced downregulation or silencing of *MGMT* inhibits its DNA repair mechanism of alkyl group removal, thereby making tumor cells sensitive to cytotoxic alkylating agents like temozolomide which improves patients’ overall survival [5, 6]. Another genetic alteration in astrocytic tumors that confers a significant survival benefit is a mutation of isocitrate dehydrogenase (*IDH*). Point mutations in the *IDH1* or *IDH2* gene are correlated with a better overall survival and while they occur frequently in lower-grade astrocytomas (WHO grade II and III) and secondary glioblastomas, they are rare (< 10%) in primary glioblastomas [7]. This genetic influence on prognosis needs to be considered when therapies in astrocytic tumors are compared or a predictive parameter like the *MGMT* promoter methylation is determined.

Currently, there is no direct method to determine the enzymatic activity of MGMT in tumor samples, which is the reason why the *MGMT* promoter methylation serves as an indirect tool. Methylation-specific PCR (MSP) [8] and pyrosequencing [9] are commonly used techniques for routine testing in molecular diagnostics of glioblastoma. While the latter is based on quantitative measurements of a handful of CpGs, MSP returns a binary decision with uncertainties in the determination of technical cutoffs [10–12]. MSP, however, is cost-effective and easy when compared to other methods, since it does not ask for special equipment beyond that present in any laboratory doing PCRs.

The positioning of PCR and sequencing primers in CpG-rich regions such as the *MGMT* promoter is challenging. Thus, it is unclear whether MSP or pyrosequencing assays, like the MGMT Pyro Kit (QIAGEN), perform in an unbiased manner. Illumina 850 K (EPIC) BeadChip microarrays are increasingly used for clinical diagnostic and prognostic estimations as they allow a genome-wide methylation profiling [13–15]. However, BeadChip arrays only cover few CpGs in promoter regions such as *MGMT* and detection is highly dependent on probe position and primer extension effects [16]. In contrast to all these selective methods, profiling by local deep bisulfite sequencing (LDBS) offers a direct and (countable) quantitative single CpG methylation readout covering regions of up to 500 bp [17]. By cautious primer design, the potential biases in LDBS are reduced to a minimum. We therefore generated LDBS data across the *MGMT* promoter region as a “gold standard” in 69 glioblastoma and 11 anaplastic astrocytoma samples. We then compared these results with the routinely obtained data from pyrosequencing (therascreen MGMT Pyro Kit, Q24, MDx) and two different MSPs [18, 19], and discuss the results with regard to their practical implementation in everyday diagnostic procedures [7].

## Results

### Bisulfite profiling of the exon 1 and intron 1 region of the *MGMT* promoter

In a glioblastoma/anaplastic astrocytoma cohort of 80 tumors, standard molecular diagnostic procedures were conducted, including *MGMT* promoter methylation analysis using two methylation-specific PCRs (MSP) [8, 18, 19] and pyrosequencing [20–22]. Following the cutoff values for the therascreen MGMT Pyro Kit as suggested by Reifenberger and colleagues, we regard an average methylation percentage larger than 8% as methylated for diagnosis [23]. For both “methylated” MSPs, any visible PCR product will signify a methylation of the *MGMT* promoter. Comparing MSP results using primer pairs published by Esteller et al. (2000) and primer pairs published by Felsberg et al. (2009), we observed concordance, i.e., presence or absence of PCR products in both MSPs, for 58 tumors, while discordant results, i.e., absence of PCR product in one of the MSPs, were obtained for 22 tumors (Additional file 1: Table S1) [18, 19]. Out of 22 discordant samples, 13 were detected as methylated using the Esteller primer pair, while 9 samples were found to be methylated by the Felsberg primer pair only. Quantitative pyrosequencing of four CpG positions in *MGMT* exon 1 revealed average methylation values between 2.5 and 70.3% for the MSP-concordant methylated tumors, between 1.5 and 16.3% for the MSP-concordant unmethylated tumors and between 1.2% and 23.5% for the discordant tumors. Among the MSP-discordant cases, five of the 13 “Esteller-methylated/Felsberg-unmethylated” (38.5%) and three of the nine “Felsberg-methylated/Esteller-unmethylated” (30%) samples were considered methylated by pyrosequencing, i.e., above a cutoff of 8% average methylation. In three of the 26 cases with concordant MSP-unmethylated results (11.5%), quantitative pyrosequencing also produced an average methylation value above 8%.

**Fig. 1:**
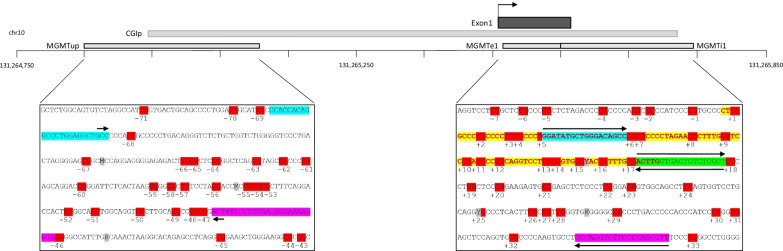
Schematic overview on the *MGMT* promoter region with positioning of exon 1 (ENSG00000170430), promoter-associated CpG island (CGIp) and sequenced amplicon regions; hg19 genomic position is given in bp; relative position of CGIp and amplicons subjected to local deep bisulfite sequencing (*MGMT*up, *MGMT*e1 and *MGMT*i1) are shown as grey bars; *MGMT*up and *MGMT*e1 forward primer sequences are highlighted in turquoise, *MGMT*up and *MGMT*i1 reverse primer sequences are highlighted in pink; the sequence used for *MGMT*i1 forward and *MGMT*e1 reverse primers is highlighted in green; arrows indicate the directionality of primers; exon1 sequence is highlighted in yellow; grey: ambiguously called nucleotides (taken from dbSNP153); red: CpG sites, numbered relative to the TSS

**Fig. 2:**
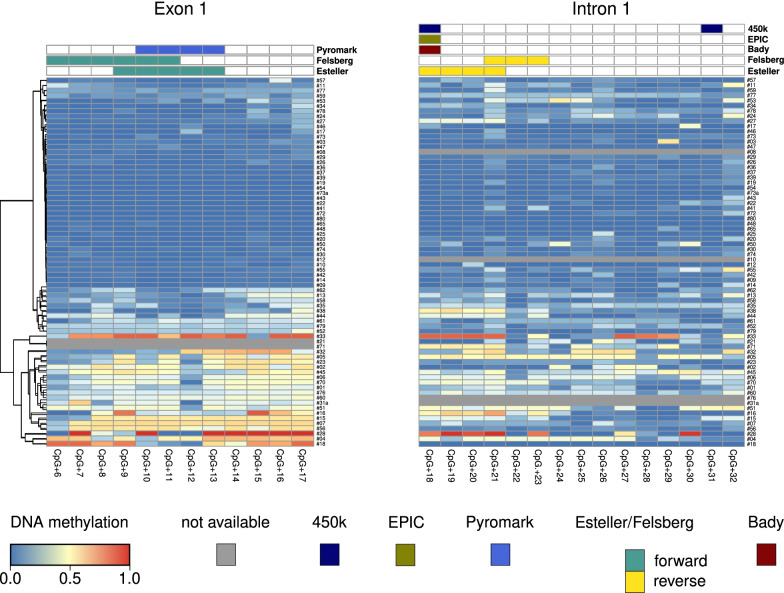
DNA methylation heatmap of all CpGs in the *MGMT*e1 (left) and *MGMT*i1 (right) amplicons in malignant astrocytoma samples; CpGs are numbered relative to the TSS; grey lines represent missing data for the respective sample; CpGs covered by pyrosequencing (PyroMark) and MSP (Felsberg and Esteller) or present on the 450 K/850 K (EPIC) BeadChip arrays are indicated at the top. Samples were clustered according to their methylation levels in *MGMT*e1 and put in the same order in *MGMT*i1 for clarity reasons

To obtain a detailed picture of the CpG methylation levels covered by MSP primers and pyrosequencing, we performed local deep bisulfite sequencing (LDBS) using oligos that amplify the exon 1/intron 1 region in a methylation-independent manner (Fig. 1) [17]. Out of 80 tumors (82 samples), we managed to obtain 72 (exon 1) and 70 (intron 1) amplicons that were subjected to LDBS, respectively. As shown in Fig. 2, sample-wise averaged *MGMT* exon 1 (*MGMT*e1) methylation levels showed an almost bimodal distribution, with unmethylated tumor samples clearly separated from methylated ones in the hierarchical clustering in exon 1.

CpG-wise, *MGMT*e1 methylation appeared to be rather homogeneous across all CpGs with CpG + 6 and CpG + 12 showing lower DNA methylation compared to all other CpG sites in the *MGMT*e1 amplicon. In *MGMT* intron 1 (*MGMT*i1), sample- and CpG-wise methylation appeared to be more heterogeneous. The overall methylation decreased from *MGMT*e1 to *MGMT*i1 with single CpGs (CpG + 21, CpG + 32) showing an increase in methylation and some (CpG + 22, CpG + 28 to + 31) showing a decrease across all samples.

**Fig. 3:**
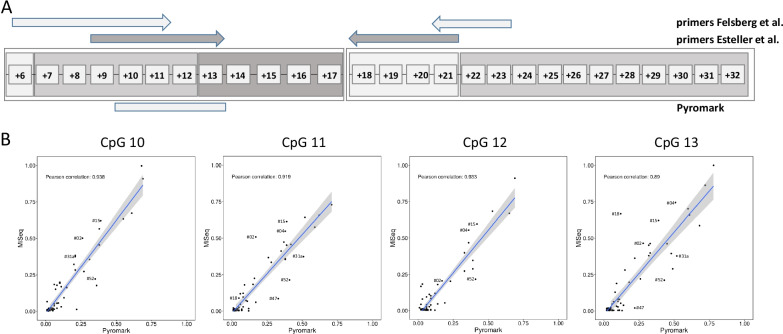
**A** Grouping of CpGs in *MGMT*e1 (CpG + 6 to CpG + 17) and *MGMT*i1 (CpG + 18 to CpG + 32) into methylation domains; CpG sites covered by MSP primers are indicated by arrows, CpGs analyzed with pyrosequencing (PyroMark) are indicated as a box at the bottom. **B** Correlation plots of absolute methylation data obtained by local deep bisulfite sequencing (MiSeq) and pyrosequencing (PyroMark) for CpGs + 10, + 11, + 12 and + 13; each dot represents the methylation level of the indicated CpG from a single sample; Pearson correlation coefficient is indicated together with trend line, confidence intervals and IDs of consistent outlier samples

**Fig. 4:**
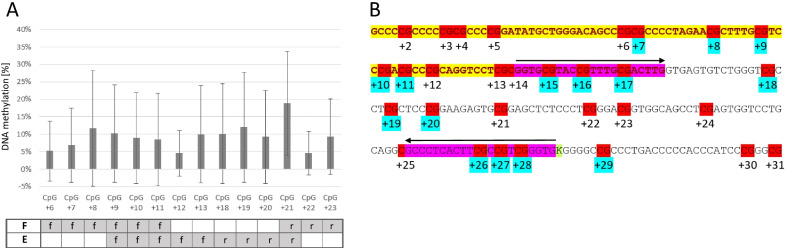
**A** Average DNA methylation of CpGs covered by MSP primers in samples discordant for MSP-based methylation detection; F, Felsberg primer set; E, Esteller primer set; f,  forward primer; r, reverse primer. **B** Proposed localization of primers for an optimized *MGMT* promoter MSP assay; yellow: exon 1; turquoise: neighboring CpGs discriminating between unmethylated and methylated states with lowest logistic regression model *p* values; pink: forward and reverse primer binding sites, arrows indicate the directionality of the primers; grey: ambiguously called nucleotides (taken from dbSNP153); red: CpG sites, numbered relative to the TSS

Taking all CpGs in *MGMT*e1 and *MGMT*i1 into account, we grouped the CpGs into five distinct methylation domains showing distinct patterns across the samples (Fig. 3A, see “Methods”). Interestingly, CpGs + 10, + 11, + 12 and CpG + 13, which are covered by the PyroMark assay, grouped into different methylation domains. Overall, the PyroMark results highly correlated with the local deep sequencing data (Pearson correlation *r* ≥ 0.89). Outlier samples were partially consistent between the single CpGs (Fig. 3B). The discordant MSP results (22 out of 80) showed an average methylation of 10.9% and 10% with bisulfite profiling for exon 1 (*MGMT*e1 and *MGMT*i1) for the solely “Esteller primer-methylated” (*n* = 13) and “Felsberg primer-methylated” (*n* = 9) samples, respectively. Looking into deep sequencing data of those CpGs within the MSP primer binding sites, we found at each CpG highly variable methylation values with an average between 4 and 12% for 13 out of 14 CpGs (Fig. 4A). CpG + 21 at the 3′-end of the Felsberg reverse primer showed an extraordinary high average methylation level of 19% and is grouped into a different methylation domain (Fig. 3A) making this oligo potentially the most unreliable one in the currently used oligo sets for routine testing.

Due to the classification into distinct methylation domains, we were able to systematically search for CpGs that reliably differentiate between methylated and unmethylated samples. Using logistic regression models, we computed *p* values for different combinations of CpGs and used this value as an indication for potentially new MSP primer designs (Fig. 4B). Lowest *p* values were obtained when forward primers were placed on CpG + 7 to CpG + 9 (*p* = 0.0016822, Additional file 2: Table S2, e1i1_domain2) and CpG + 15 to CpG + 17 (*p* = 0.0104365, Additional file 2: Table S2, e1i1_domain3). CpG + 10 and CpG + 11 together provided one of the lowest *p* values (*p* = 0.000128); however, the combined CpG + 7 to CpG + 11 (*p* = 0.003051) sequence stretch performed worse in comparison with CpG + 7 to CpG + 9 alone. The most significant CpGs for the binding of reverse primers could be found encompassing CpG + 18 to CpG + 20 (*p* = 0.006266, Additional file 2: Table S2, e1i1_domain4) and CpG + 26 to CpG + 29 (*p* = 0.0002321, Additional file 2: Table S2, e1i1_domain5). Because of an annotated common SNP (minor allele frequency > 1%) between CpG + 28 and CpG + 29, we recommend CpG + 26 to CpG + 28 as potential primer binding sites (*p* = 0.000777, Additional file 2: Table S2, e1i1_domain5).

When testing different MSP primer pairs, the best performance was obtained with the forward primer covering CpG + 15 to CpG + 17 and the reverse primer covering CpG + 26 to CpG + 28 (Fig. 4B). In fact, MSP on unmethylated peripheral blood (PBL) DNA and methylated EpiTect Control DNA (Qiagen) revealed the discriminative power of the proposed primer pair (Additional file 3: Fig. S1). Because of limited sample material, only 23 GBMs could be analyzed with the new primers resulting in 20/24 and 17/20 (83% and 85%) concordant results with pyrosequencing and LDBS, respectively (Additional file 1: Table S1 “Tierling MSP”).

### Investigation of DNA methylation upstream of the *MGMT* promoter

Since the prominent promoter CpG island (CGIp, Fig. 1) extends 5′ beyond the so far analyzed region, we extended our analysis to a sequence section − 605 to − 353 bp relative to the transcriptional start site (TSS), called *MGMT*up, covering the 5′-portion of the CGIp (Fig. 1). Because of high CpG density and homopolymer stretches, the region between − 353 bp and the TSS was not amplifiable. In *MGMT*up, CpG-wise and sample-wise methylation were very heterogeneous. With the exception of samples #02, #07, #33 and #60, DNA methylation increased from a mosaic pattern (CpG − 47 to CpG − 61) towards highly methylated CpGs (CpG − 68 to CpG − 62) with increasing distance to the TSS (Additional file 4: Fig. S2a). Grouping *MGMT*up into different methylation domains similar to *MGMT*e1 and *MGMT*i1 revealed five different domains with two domains consisting of single CpGs (CpG − 67 and CpG − 68) (Additional file 4: Fig. S2a). CpGs with the highest discriminative power between lowly methylated (59) and highly methylated (13) samples were found in domain 5 (*p* < 7.74 × 10^–5^), namely CpG − 48 (10.07% ± 17% vs. 64.29% ± 32%), CpG − 52 (13.44% ± 21% vs. 60.63% ± 28%), CpG − 56 (17.85% ± 23% vs. 65.95% ± 20%), CpG − 49 (12.76% ± 19% vs. 53.28% ± 29%) and CpG − 51 (11.3% ± 19% vs. 56.22% ± 29%). Because of the rather high methylation level in the 59 lowly methylated samples, a diagnostic MSP assay with a positive/negative readout seems not to be feasible. Alternatively, quantitative methods like primer extension, qPCR or pyrosequencing assays focusing on CpG − 48 to CpG − 56 could be used. Comparing sample methylation levels in *MGMT*e1 with the other regions showed that lowly (< 9%), intermediately (9–30%) and highly methylated (> 30%) samples were, in tendency, proportionally higher methylated in *MGMT*up in comparison with *MGMT*e1 and *MGMT*i1 (Additional file 4: Fig. S2b). Interestingly, we observed a slight increase in methylation in *MGMT*i1 for the lowly methylated sample group (defined on the *MGMT*e1 methylation state) and a decrease for the intermediately and highly methylated sample group. This observation points to transcriptional downregulation of *MGMT* in glioblastoma being the result of higher activity of DNA methyltransferases in *MGMT*up and *MGMT*e1 compared to *MGMT*i1.

### Association of CpG-wise methylation calls with progression-free survival

It was described that CpG methylation in the *MGMT* promoter region can be predictive for therapy outcome coupled with overall survival of glioblastoma patients [5, 6, 24–28]. Based on local deep bisulfite sequencing data, we performed Cox regression analysis on 50 samples (GBM without *IDH* mutation) excluding #18 and #23 which we regard as outliers because of their high progression-free survival (PFS, Additional file 1: Table S1). We did not find any CpG in *MGMT*e1 or *MGMT*i1 that significantly (*p* < 0.05) or in tendency (*p* < 0.1) correlated with progression-free survival. In *MGMT*up, we found CpG − 48, CpG − 61 and CpG − 64 close to *p* = 0.01 (Additional file 5: Table S3).

Using the average of all three CpGs as input to the Cox regression model, we found patients with low *MGMT*up methylation with significantly improved progression-free survival (PFS) (*p* = 0.04379) (Additional file 6: Fig. S3a). Analyzing 450 K data of 93 samples from The Cancer Genome Atlas (TCGA, data set “glioblastoma multiforme” excluding IDH1 mutants), we could not confirm any prognostic value of CpG − 48 which is represented as cg01341123 (*p* = 0.9056) (Additional file 6: Fig. S3b, for location compare Additional file 4: Fig. S2a). CpG − 47, CpG − 49 and CpG − 50, present on the array as cg25946389, cg23998405 and cg02022136, did also not show any predictive power with respect to progression-free survival (*p* = 0.6964, *p* = 0.8063 and *p* = 0.3582), respectively (data not shown, for location compare Additional file 4: Fig. S2a). Finally, we took a closer look on the methylation of CpG + 18 (cg12981137, compared Fig. 2), which was previously found by a logistic regression model to be predictive (together with cg12434587, which corresponds to CpG − 36, not present within our amplicons) for treatment outcome and prognosis in a test cohort of 63 glioblastomas and 450 K data taken from TCGA [13]. Neither local deep sequencing data of the here presented cohort nor the 450 K data set downloaded from TCGA could confirm the predictive quality of cg12981137 alone (*p* = 0.8671 and *p* = 0.9025, respectively, Additional file 6: Fig. S3c, d). We cannot exclude that adding local deep sequencing data of cg12434587 would enhance prognostic significance but at least doubt the informative power of cg12981137 as exclusive methylation marker in routine testing. Additionally, Bady and colleagues correlated the methylation status with the overall survival, while we used, due to limited OS data availability, progression-free intervals for a logistic regression [13]. While these two parameters are often correlated [18, 19], they do not necessarily show the same trend [20, 29].

## Discussion

In this study, we characterized methylation of single CpG sites of the *MGMT* promoter in tumor samples from first neurosurgical intervention in a cohort of 69 glioblastoma multiforme (WHO grade IV) and 11 anaplastic astrocytoma (WHO grade III) patients, who subsequently received radiotherapy and temozolomide treatment. The transition from astrocytoma WHO grade III to glioblastoma multiforme often occurs as a smooth transition, so the classification as a WHO grade III astrocytic tumor may in some cases be due to a sampling error. In contrast to astrocytoma WHO grade II, astrocytic tumors WHO grade III and IV have a similarly high mitotic activity.

By using local deep bisulfite sequencing, we showed that for *MGMT*e1 DNA methylation distributed almost binary among the samples, a phenomenon already observed previously [30]. In *MGMT*i1 and *MGMT*up, methylation distribution was much more heterogeneous across samples and CpG sites. Overall, single CpG methylation levels tended to increase with distance to the TSS, an effect known for active genes and tumor suppressors in cancer [31, 32]. The detailed CpG-wise data collection enabled us to compare commonly used diagnostic assays such as pyrosequencing and MSP, with local deep bisulfite sequencing as the gold standard [16, 33], thus revealing that pyrosequencing data obtained with the PyroMark Q24 assay was highly correlated with local deep bisulfite sequencing results. This is in concordance with earlier studies confirming that pyrosequencing provides reliable quantitative and sensitive results in *MGMT* methylation detection [34–38]. However, this leaves the difficulty to decide on a cutoff value, i.e., for the average methylation of a limited number of CpGs in the *MGMT* promoter that should ideally reflect on the MGMT activity in the tumor, which is until now rather difficult to obtain. As clinically relevant cutoff, seven, eight and ten percent average methylation of CpGs analyzed by pyrosequencing have been suggested [39–41]. For the integrated neuropathological diagnosis, we usually follow the 8% cutoff recommended by Reifenberger et al. (2012) in concordance with the results obtained by both MSPs [41]. For local deep bisulfite sequencing, relevant cutoffs still need to be determined, which would require a larger study cohort. Yet, even with a suitable cutoff most neurosurgery/neuropathology laboratories, especially smaller ones, will probably not implement this method, due to a cost–benefit calculation and, even more importantly, the fact that collecting samples for one sequencing run would not comply with the delivery of results within a few workdays.

The question with any method to determine *MGMT* promoter methylation is whether the result really serves the patient, i.e., from what percentage of methylation on does the patient actually profit from a therapy with alkylating agents justifying the side effects. This applies especially in elderly patients and whenever an alternative treatment can be taken into consideration. Methylation-specific PCRs (MSPs) seem to produce a certain amount of unmethylated results that show methylation with quantitative methods (three cases in our cohort). One of our practical approaches to reduce false negatives, yet obtain a fast result during routine diagnostics, is the application of two MSP primer-sets on each sample. When both MSPs provide a methylated PCR product, we consider the sample as methylated. If one or both MSP primer pairs fail to produce a methylated PCR product, pyrosequencing immediately follows as the decisive factor for the *MGMT* promoter status. According to an international inter-laboratory study by Reifenberger et al. (2014), MSP was the most commonly used method for *MGMT* methylation status determination [41]. In our opinion, it still proves to be a cost-effective and comparatively easy method when being part of a diagnostic scheme with more than one method.

Comparing MSP results to local deep bisulfite sequencing revealed that primer design is crucial to reach sufficient sensitivity and specificity to reduce the number of false negative PCR results [42–45]. To avoid unreliable primer binding as suspected, e.g., the Felsberg reverse primer, we grouped CpG dinucleotides with similarly low or high methylation into methylation domains to select neighboring and discriminative CpGs within the same methylation domain. As a result, we proposed subsets of CpGs that could be part of optimized MSP primers in *MGMT*e1/i1 and for quantitative methylation assays like pyrosequencing, MethyLight [46] or single-nucleotide primer extension [47]. In addition, bisulfite profiling of *MGMT*up revealed discriminative CpG sites that could potentially be used in separate or supplementary quantitative assays to improve diagnostic and/or predictive power. Experimental testing of different primer combinations resulted in a new MSP primer pair with improved performance on a subset of our cohort. Application on larger independent cohorts will show if there is a benefit of new or adapted assays in diagnostic and/or predictive estimations.

Correlating patient’s clinical data to local deep bisulfite sequencing results showed that none of the CpGs in *MGMT*e1 and *MGMT*i1 was predictive for the duration of a progression-free survival in our sample cohort of primary glioblastoma without *IDH* mutations and treatment with radiotherapy and concomitant chemotherapy with temozolomide. This is in contrast to previous meta-analyses [28, 48, 49] and other recent studies [50–53]. However, studies with nonsignificant effect on survival data also exist [54, 55]. This may largely depend on how the progression-free survival is defined and detected. Routinely performed neuroimaging without neurological symptoms in set intervals may prepone the noted date of tumor remission in comparison with an onset of clinical aggravation. The actual location of the tumor in the brain, however, is crucial for the beginning of neurological symptoms. Massive tumor growth in an inconspicuous location may be overlooked for some time and artificially prolong the noted progression-free interval. In the present cohort, we had hardly any data on the overall survival of the patients. Also, the size of the cohort, immune cell composition, hot spot mutations or genomic aberrations within the group of glioblastoma multiforme may influence the outcome of survival analyses [56–59], possibly making them inconclusive.

We also found CpG + 18 methylation not to be meaningful towards prognosis in three different independent glioblastoma cohorts, which suggests that combinatorial analysis as shown by Bady and colleagues might improve survival analysis and biomarker detection significantly [13, 60]. In fact, for *MGMT*up we found three CpGs, which in combination showed significant correlation with PFS depending on their methylation level. We could not reveal a predictive effect of any of the single CpG methylation calls in our cohort. The predictive value of those CpGs needs to be evaluated in independent cohorts.

## Conclusion

Taken together, our study shows that quantitative assays, like local deep bisulfite sequencing or pyrosequencing, provide reliable quantitative data of *MGMT* promoter methylation. We found a high correlation of bisulfite profiling with pyrosequencing. For pyrosequencing, cutoff values have been defined in other studies, while for local deep bisulfite sequencing relevant cutoffs still need to be determined using larger study cohorts. MSP data generation is fast and cost-effective, but CpGs, covered by the widely used Esteller and Felsberg MSP primers, group into different methylation domains, which results in significant numbers of negative MSP results that prove to be methylated with quantitative methods. In practice, a scheme involving more than one method and/or at least two primer pairs will improve the accuracy of the result. Together with bisulfite sequencing data of a newly identified region upstream of the *MGMT* promoter, *MGMT*up, we found neighboring CpGs within the same methylation domain being highly discriminative between unmethylated and methylated sequences, which were used to design a new MSP primer set with improved performance. Testing this primer set on larger sample cohorts will prove its suitability in routine diagnostics in the future.

## Methods

### Tumor sample cohort and DNA extraction

Tumor samples had been taken during the first surgical removal of the tumor (all but two in the department for Neurosurgery, Saarland University Medical Center in Homburg) and had been subsequently fixed in 4% buffered formaldehyde and embedded in paraffin for routine diagnostics. After the neuropathological diagnosis, we used the corresponding hematoxylin-/eosin-stained slide as reference to the 10-µm-thick FFPE sections for DNA preparation to choose tumor tissue parts without necrotic areas and isolated DNA using the QIAamp DNA Micro Kit (#56304, QIAGEN, Hilden, Germany) according to the manufacturer’s instructions. DNA yield and quality were determined using a NanoPhotometer® N60 (Implem GmBH, Munich, Germany). We admitted only patients with an astrocytic tumor grade III or IV that underwent the Stupp protocol [2], i.e., concomitant radiotherapy and temozolomide administration following surgery to our retrospective study. The study cohort includes 68 glioblastoma multiforme without proven *IDH* mutations, one glioblastoma with an R132C mutation and eleven anaplastic astrocytomas (grade III) with two having an *IDH1* mutation (R132H and R132C) (see Additional file 1: Table S1). We determined the progression-free survival (PFS) by either neurological symptoms attributed to tumor remission or neuroimaging showing tumor recurrence. All relevant data regarding treatment and recrudescence were extracted from the patients’ records at the Saarland University Medical Center in Homburg (Saar), Germany. For subsequent analysis, we blinded all samples and kept only the mere necessary data such as progression-free survival (PFS), age at onset, gender and year of birth as shown in Additional file 1: Table S1 together with results for all applied methods. The project was approved by the ethics committee from the Aerztekammer des Saarlandes (No. 133/20).

### Methylation-specific PCR (MSP) and Pyrosequencing

For MSP and pyrosequencing, we used 40 ng genomic DNA for bisulfite conversion using the EpiTect Fast Bisulfite Kit (#59824, QIAGEN, Hilden, Germany) according to manufacturer’s instructions. For each batch of samples, 20 ng of unmethylated human control DNA was also subjected to bisulfite conversion and the respective PCRs, in addition to bisulfite negative and positive bisulfite-treated controls (#59695 EpiTect PCR Control DNA Set, QIAGEN, Hilden) and non-template controls (Aqua bidest.).

To detect methylated and unmethylated bisulfite-converted DNA, we used two different primer sets. The first set, published by Esteller et al. (2000), amplifies an unmethylated fragment of 93 bp (primer sequences forward 5'-TTTGTGTTTTGATGTTTGTAGGTTTTTGT-3' and reverse 5'-AACTCCACACTCTTCCAAAAACAAAACA-3') and a methylated fragment of 89 bp (forward 5'-TTTCGACGTTCGTAGGTTTTCGC-3' and reverse 5´-GCACTCTTCCGAAAACGAAACG-3') [18]. The second set, published by Felsberg et al. (2009), amplifies an unmethylated fragment of 129 bp (primer sequences forward 5′-TGTGTTTTAGAATGTTTTGTGTTTTGAT-3′ and reverse 5′-CTACCACCATCCCAAAAAAAAACTCCA-3′) and a methylated fragment of 120 bp (primer sequences forward 5′-GTTTTTAGAACGTTTTGCGTTTCGAC-3′ and reverse 5′-CACCGTCCCGAAAAAAAACTCCG-3′) [19]. Our proposed MSP primer pair amplifies an unmethylated/methylated fragment of 126 bp (primer sequences forward 5′-GGTG^C^/_T_GTAT^C^/_T_GTTTG^C^/_T_GATTTG-3′ and reverse 5′-CACCC^G^/_A_AC^G^/_A_AC^G^/_A_AAATAAAAAC-3′) using GoTaq Hot Start Green Master Mix (Promega) and 10 pmol of each primer in a 25 µl reaction. After an initial denaturation at 95° C for 2 min, 35 cycles of 95° C 45 s, 57° C 45 s and 72° C 30 s were applied with a final extension at 72° C for 5 min. The resulting PCR products were visualized with the LONZA FlashGel system using 2.2% gels (Flash Gel™ DNA cassettes, #57032, Biozym Scientific GmbH, Germany), a Flash Gel™ DNA marker (50–1.5 kb #57033) with the included camera for documentation. MSP results for the individual cases are documented in Additional file 1: Table S1 (0 = unmethylated; 1 = methylated).

For pyrosequencing, we applied the therascreen MGMT Pyro Kit (#971,061, QIAGEN, Hilden, Germany) using the PyroMark Q24 MDx according to manufacturer’s instructions and used the controls described above.

We determined isocitrate dehydrogenase (*IDH*) mutations for *IDH1* (*Arg 132*) and *IDH2* (*Arg172*) using the PyroMark Q24 MDx and a custom assay based on the publication by Thon et al. (2012) [61]. In brief, we used reagents from the PyroMark Gold Q24 Reagents (#970,802, QIAGEN, Hilden, Germany) according to manufacturer’s instructions with primers obtained from QIAGEN (IDH1 forward primer biotinylated 5′-biotin-GAAATCACCAAATGGCACCATAC-3′, reverse primer 5′-TTGCCAACATGACTTACTTGATCC-3′ and sequencing primer 5′-TGATCCCCATAAGCAT-3′; IDH2 forward primer biotinylated 5′-CATCCTGGGGGGGACTGT-3′, reverse primer 5′-ACCCTGGCCTACCTGGTCG-3′ and sequencing primer 5′-AGCCCATCACCATTG-3′).

### Bisulfite amplicon preparation and sequencing

DNA obtained from the FFPE tissue sections was bisulfite-converted using the EpiTect Bisulfite Kit (QIAGEN, Hilden, Germany) or the EZ DNA Methylation Gold Kit (Zymo Res., Irvine, CA). Subsequently, three different PCRs were performed (4 µl bisulfite-treated DNA, 80 mM Tris–HCL, 20 mM (NH_4_)_2_SO_4_, 0.2% Tween-20, 2.5 mM MgCl_2_, 0.2 mM of each dNTP, 2.5U HotFirePol (Solis BioDyne, Tartu, Estonia)) using 250 pM of each primer (*MGMT*up: 5′-TTATTATAGGTTTTGGAGGTTGTT-3′, 5′-TACCTTTTCCTATCACAAAAATAAT-3′; *MGMT*e1: 5′-GGATATGTTGGGATAGTT-3′, 5′-ACCCAAACACTCACCAAAT-3′; *MGMT*i1: 5′-GATTTGGTGAGTGTTTGGGT-3′, 5′-AAACTAAACAACACCTAAA-3′) with Illumina universal adapter sequences attached at the 5′-end. To improve amplification efficiency, we added 0.2 µl Hot Start-IT Binding Protein (Thermo Scientific) to the *MGMT*up and *MGMT*i1 PCR reactions. PCRs were performed in a thermocycler starting with 15 min 95° C followed by 45 cycles 95° C 60 s, 54° C 75 s, 72° C 90 s and a 10-min final extension at 72° C. We purified amplicons using Agencourt AMPure XP beads (Beckman Coulter, Krefeld, Germany), and diluted, pooled and sequenced (v3 chemistry: 2 × 300 bp paired-end) them on the Illumina MiSeq following the manufacturer’s instructions.

### Data evaluation and statistics

Sequencing reads were aligned to the reference sequence (*Homo sapiens* genome build GRCh37/hg19) and quality-filtered using the BiQ Analyzer HT program [62]. We conducted additional data analysis using custom R scripts (available at https://github.com/schmic05/MGMT_methylation) based on the aligned sequencing reads or the BiQ Analyzer HT output. Average DNA methylation levels for the CpGs were computed across the sequencing reads using BiQ Analyzer HT and used for visualization as methylation heatmaps. The samples were hierarchically clustered using the Euclidean distance and complete linkage on the CpG methylation values in exon 1.

We defined methylation domains in the amplicon sequencing data as adjacent CpGs that behave similarly across the samples within the amplicon. To that end, we first computed the mean and variance of all CpGs across the samples. Based on these values, we computed the difference between the mean and the variance between any two adjacent CpGs. According to the distribution of the differences in mean and variance of the methylation values, we defined the 90% quantiles as the threshold, where we set the border of each of the methylation domains for the three amplicons (*MGMT*up, *MGMT*e1, *MGMT*i1).

Based on the classification into these domains, we aimed to select those CpGs that reliably differentiate between the highly methylated and lowly methylated samples. First, we calculated the average DNA methylation value for all CpGs and samples within each of the domains. We termed those domains methylated that showed an average DNA methylation level of more than 50% and the others unmethylated. In the next step, we computed sample-wise average DNA methylation values for each of the domains individually and termed those samples as outliers per domain that showed an average DNA methylation more than two standard errors away from the mean. For the *MGMT*up region, we found higher differences across the samples and thus used four standard errors as the cutoff. For the unmethylated domains, methylated samples were termed outliers and vice versa. The information for each of the samples (outlier sample = 1, other samples = 0) was used as the output variable in a logistic regression model. We used the CpG-wise methylation values and all potential combinations of the CpG-wise methylation values as input variables in the regression models. Finally, we interpreted the *p* value of the individual logistic regression models (for each combination of CpGs) as an indicator of the discriminative power of these CpG-wise methylation states.

To determine CpGs that were associated with progression-free survival (PFS), we used the *coxph() function* from the survival R package [63] for all individual CpGs within the *MGMT*up, *MGMT*e1 and *MGMT*i1 amplicon. We used age and sex of the individuals as covariates.

## Supplementary Information

**Additional file 1: Table S1**. Glioblastoma sample cohort with clinical monitoring data (blue), MSP (orange), PyroMark (yellow) and local deep sequencing (green) results; For MSP results, 1 equals the presence and 0 the absence of a methylated PCR product, respectively. Missing results are indicated with “-”. Discordant MSP results (Esteller vs. Felsberg primer pair; proposed primer pair vs. pyrosequencing or LDBS) are highlighted in light orange. Results obtained from the same tumor but two different DNA isolations are highlighted in grey. ø=average methylation.

**Additional file 2: Table S2.** Average DNA methylation values, variances and p values for single CpGs and combinations of CpGs based on a logistic regression model; CpGs were analyzed methylation domain-wise, most significant neighboring CpGs, PyroMark and MSP-covered CpGs are highlighted in yellow, blue and red, respectively.

**Additional file 3: Fig. S1.** MSP on human PBL and EpiTect Control methylated bisulfite-treated DNA with proposed primer pairs designed based on methylation domain modelling; electrophoretic separation of MSP reactions on a LONZA FlashGel system using 2.2% gels (Flash GelTM DNA cassettes, #57032, Biozym Scientific GmbH, Germany); Marker = Flash GelTM DNA marker (50bp–1.5kb #57033), PBL = peripheral blood leukocytes, bisulfite-converted DNA, EpiTect = EpiTect Control DNA (human), methylated and bisulfite-converted (Qiagen #59655); NTC = no template control.

**Additional file 4: Fig. S2.** (A) DNA methylation heatmap of all CpGs in the *MGMT*up amplicon in malignant astrocytoma samples; CpGs are numbered relative to the TSS; grey lines represent missing data for the respective sample; CpGs present on the 450K/850K(EPIC) BeadChip arrays are indicated on top. CpGs grouped into five methylation domains (D1-D5) are highlighted in different colors with D1 and D2 represented by single CpGs (CpG -68 and CpG -67). Samples were ordered according to the sample clustering in *MGMT*e1 for clarity reasons. (B) Scatter plot with trend lines and confidence intervals based on local regression analysis; each dot represents the averaged DNA methylation of a sample per sequenced amplicon. Dots are colored based on the average methylation state in the exon 1 region: red=highly methylated (>30%), purple=intermediately methylated (between 9% and 30%), blue= lowly methylated (<9%).

**Additional file 5: Table S3.** *P* values obtained for Cox regression survival analysis for each CpG in *MGMT*up, *MGMT*e1 and *MGMT*i1; 1st sheet: all samples; 2nd sheet: statistical outliers #18 and #23 were excluded from analysis.

**Additional file 6: Fig. S3.** Kaplan–Meier survival plots including *p* values obtained from Cox regression model analysis and confidence intervals (blue/red); (A) average DNA methylation of CpG -48, CpG -61 and CpG -64, (B) TCGA 450K array-based DNA methylation of CpG -48 (cg01341123), (C) DNA methylation of CpG +18 in the presented cohort, (D) TCGA 450K array-based DNA methylation of CpG +18 (cg12981137).

## Data Availability

The data sets generated and/or analyzed during the current study and corresponding R scripts are available in the GitHub repository (https://github.com/schmic05/MGMT_methylation).
