# Supplementary figures and images for: Bisulfite profiling of the MGMT promoter and comparison with routine testing in glioblastoma diagnostics
Source: Clin Epigenetics. 2022 Feb 18;14:26. doi: 10.1186/s13148-022-01244-4 (PMC8857788; doi:10.1186/s13148-022-01244-4)

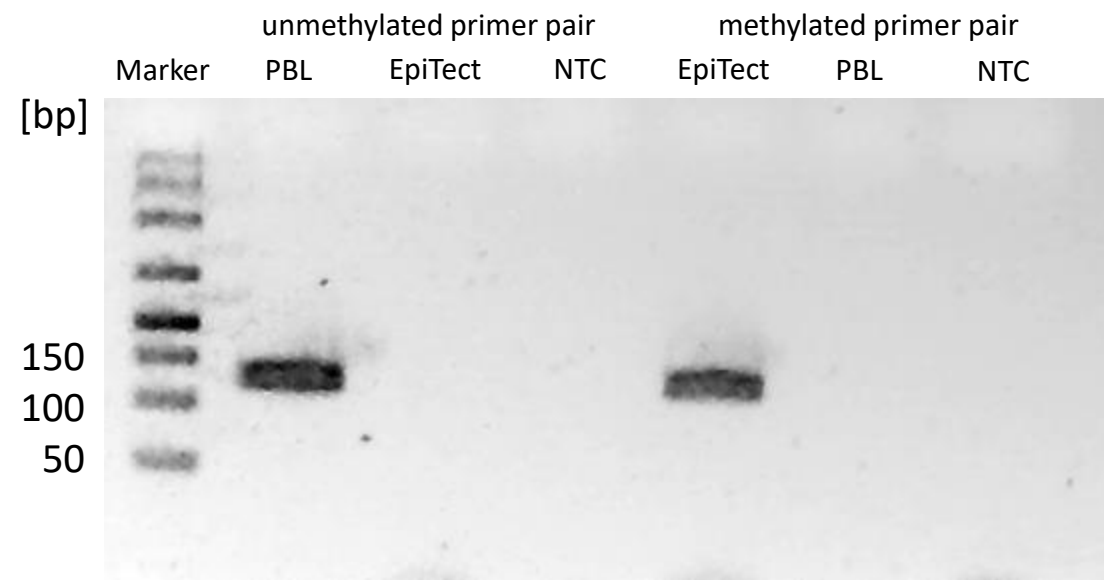

Figure S1

Supplement: Supplementary file 3 — Additional file 3: Fig. S1. MSP on human PBL and EpiTect Control methylated bisulfite-treated DNA with proposed primer pairs designed based on methylation domain modelling; electrophoretic separation of MSP reactions on a LONZA FlashGel system using 2.2% gels (Flash GelTM DNA cassettes, #57032, Biozym Scientific GmbH, Germany); Marker = Flash GelTM DNA marker (50bp–1.5kb #57033), PBL = peripheral blood leukocytes, bisulfite-converted DNA, EpiTect = EpiTect Control DNA (human), methylated and bisulfite-converted (Qiagen #59655); NTC = no template control. [file 13148_2022_1244_MOESM3_ESM.pdf]

A

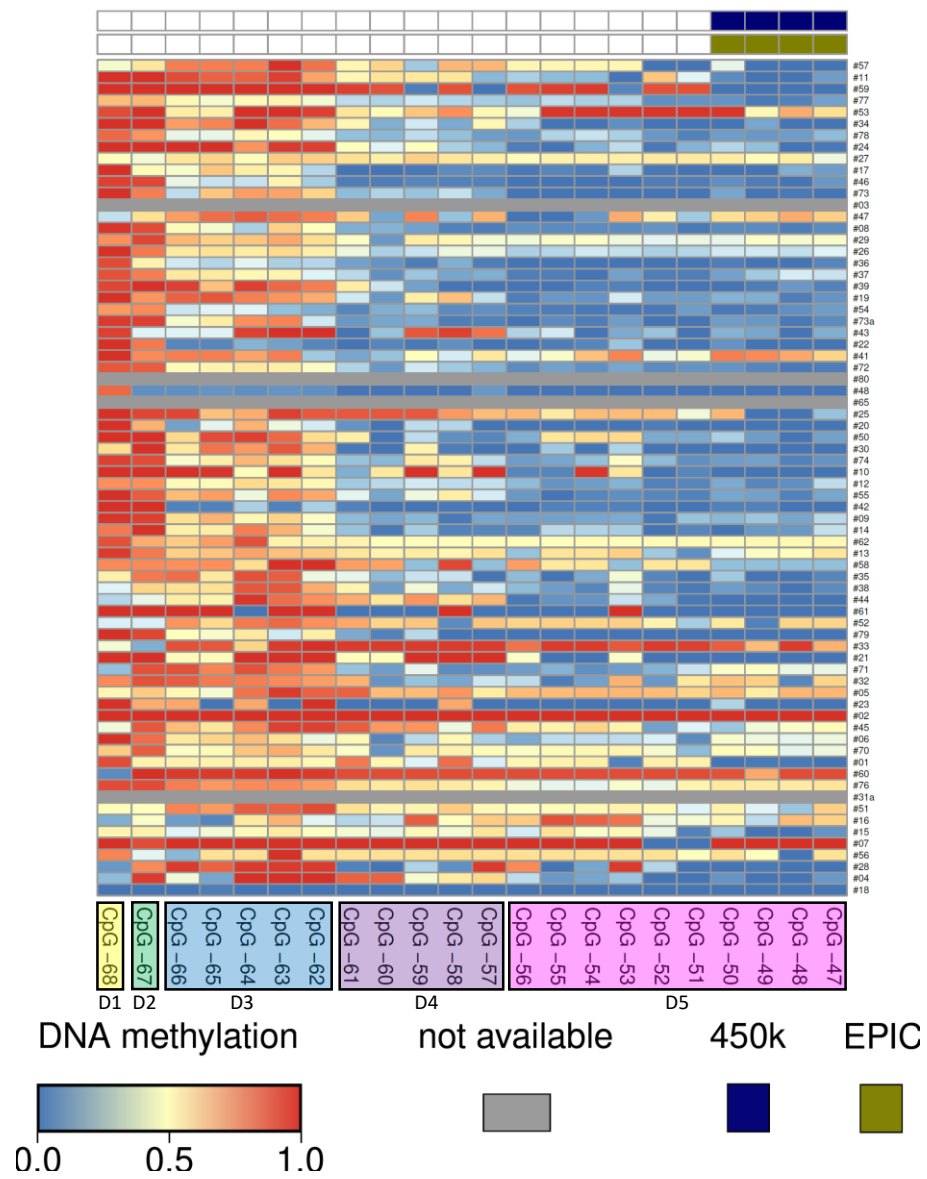

B

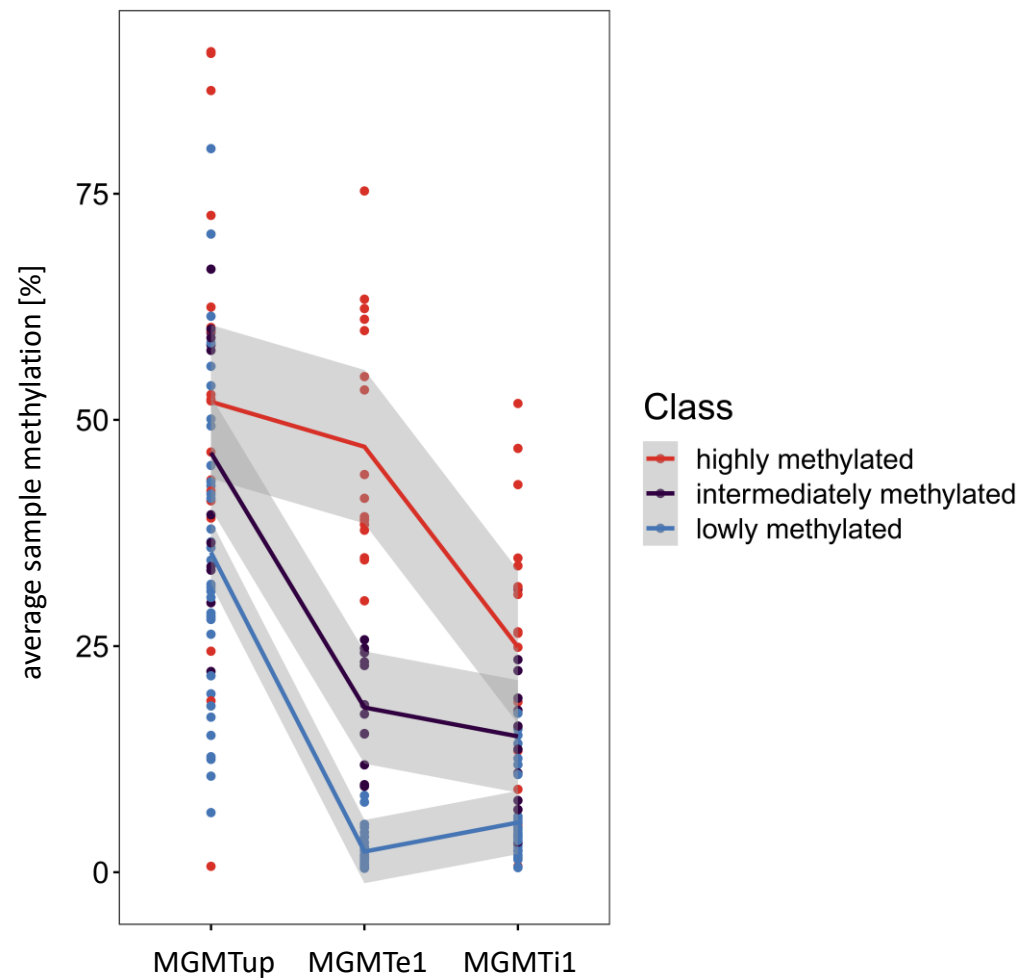

Figure S2

Supplement: Supplementary file 4 — Additional file 4: Fig. S2. (A) DNA methylation heatmap of all CpGs in the MGMTup amplicon in malignant astrocytoma samples; CpGs are numbered relative to the TSS; grey lines represent missing data for the respective sample; CpGs present on the 450K/850K(EPIC) BeadChip arrays are indicated on top. CpGs grouped into five methylation domains (D1-D5) are highlighted in different colors with D1 and D2 represented by single CpGs (CpG -68 and CpG -67). Samples were ordered according to the sample clustering in MGMTe1 for clarity reasons. (B) Scatter plot with trend lines and confidence intervals based on local regression analysis; each dot represents the averaged DNA methylation of a sample per sequenced amplicon. Dots are colored based on the average methylation state in the exon 1 region: red=highly methylated (>30%), purple=intermediately methylated (between 9% and 30%), blue= lowly methylated (<9%). [file 13148_2022_1244_MOESM4_ESM.pdf]

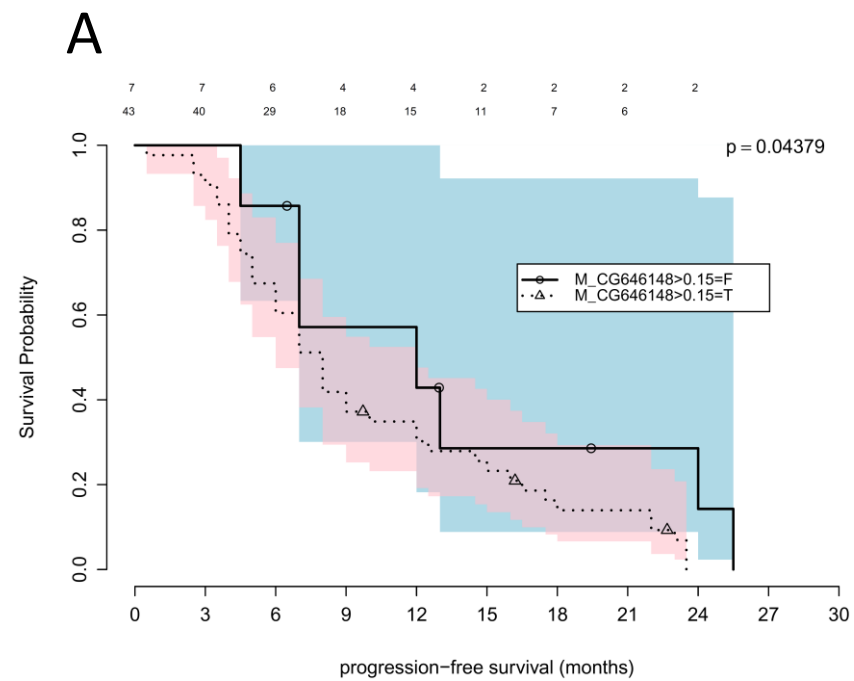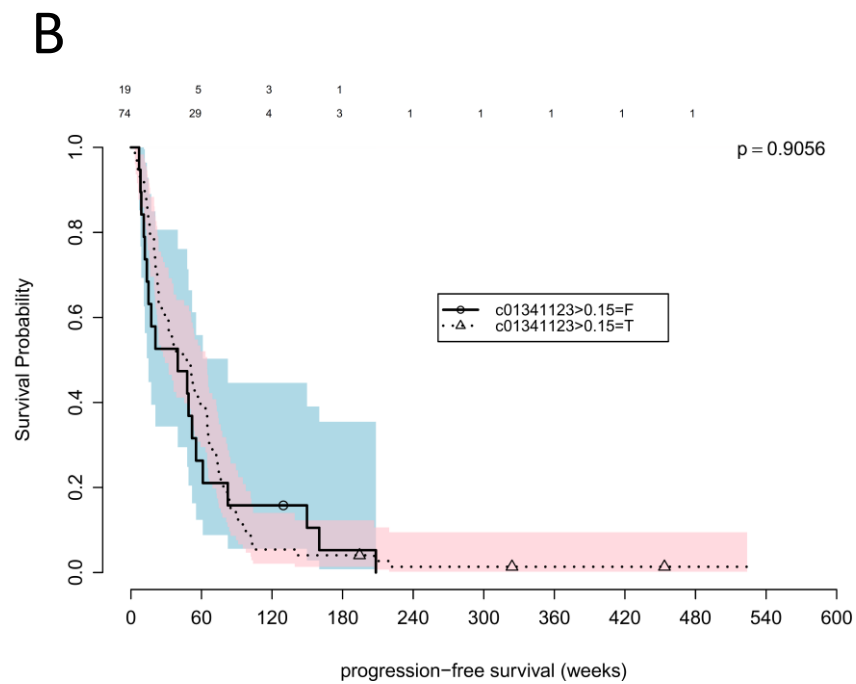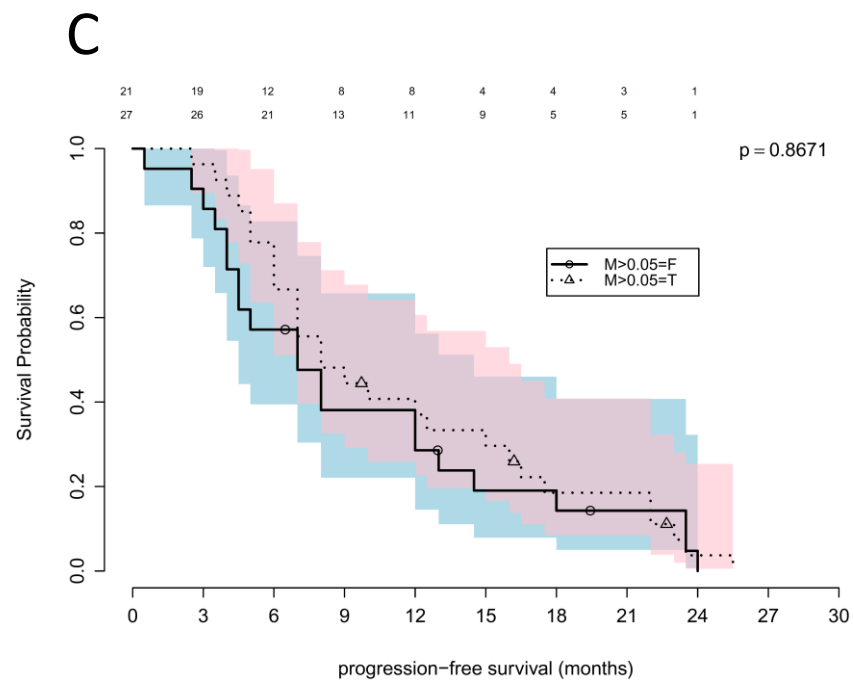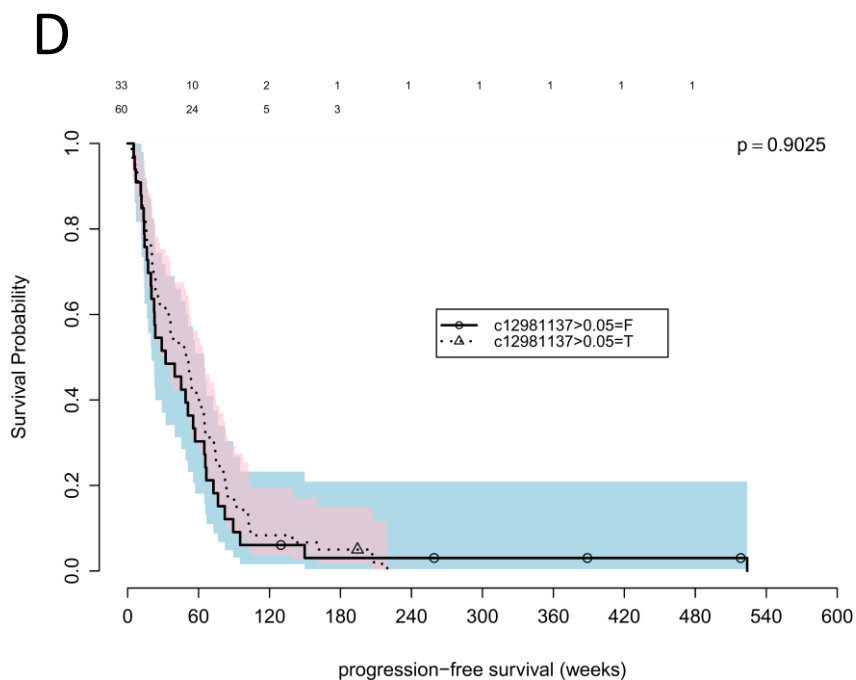

Figure S3

Supplement: Supplementary file 6 — Additional file 6: Fig. S3. Kaplan–Meier survival plots including p values obtained from Cox regression model analysis and confidence intervals (blue/red); (A) average DNA methylation of CpG -48, CpG -61 and CpG -64, (B) TCGA 450K array-based DNA methylation of CpG -48 (cg01341123), (C) DNA methylation of CpG +18 in the presented cohort, (D) TCGA 450K array-based DNA methylation of CpG +18 (cg12981137). [file 13148_2022_1244_MOESM6_ESM.pdf]
